# Reassessing the Relationship Between Exhaled Nitric Oxide and Acute Bronchodilation in Asthma Patients

**DOI:** 10.1111/cea.70156

**Published:** 2025-10-21

**Authors:** Martin Färdig, Alain Michils, Renaud Louis, Mare Sabbe, Alain Van Muylem, Florence Schleich, Andrei Malinovschi

**Affiliations:** ^1^ Department of Medical Sciences, Clinical Physiology Uppsala University Uppsala Sweden; ^2^ Chest Department Erasme University Hospital, Université Libre de Bruxelles Brussels Belgium; ^3^ Department of Pneumology University Hospital of Liège, GIGAI3, University of Liège Liège Belgium; ^4^ Department of Pneumology University Hospital of Liège, GIGAI3, Exercise Physiology Lab, University of Liège Liège Belgium

**Keywords:** asthma, bronchodilation, clinical characteristics, fraction of exhaled nitric oxide

**Summary**

- Three post‐BD Fe_NO_ profiles were observed; Fe_NO_ = (≤ −10% and ≤ +10%), Fe_NO_+ (> +10%) and Fe_NO_– (> −10%)
- The Fe_NO_– profile related to infrequent bronchodilator use, indicating treatment‐naïvety or a milder asthma phenotype

To the Editor,

Fractional exhaled nitric oxide (Fe_NO_) is an established marker of type‐2 airway inflammation valuable in asthma management [1]. However, non‐inflammatory factors, such as changes in airway calibre, may also influence Fe_NO_ levels. Airway narrowing reduces Fe_NO_ levels, likely due to reduced epithelial surface area limiting the NO diffusion from the airway lining into the lumen [2]. This effect may even be more pronounced when peripheral airways are involved, as the majority of NO is produced in the conductive airways [3]. By studying the dynamics of Fe_NO_ from pre‐bronchodilator (BD) to post‐BD, Michils et al. previously identified three distinct post‐BD Fe_NO_ profiles in asthma: a change ≤ ±10% (Fe_NO_ =), an increase > +10% (Fe_NO_+) and a decrease exceeding > −10% (Fe_NO_–), suggesting that Fe_NO_ may help locate the site of airway obstruction [4].

Therefore, in a larger population of unselected asthmatics with stricter BD‐withhold, we studied whether the post‐BD Fe_NO_ =, Fe_NO_+ and Fe_NO_– profiles could be confirmed, and secondly, if they could be associated with clinical features.

This cross‐sectional analysis included 236 asthmatics (63% females), aged 14–83 years, from the asthma clinic of Liège University Hospital (Belgium). Fe_NO_ and dynamic spirometry were performed according to current international guidelines, measured sequentially, both before and 15 min after the inhalation of BD (400 μg of salbutamol), using the NIOX Vero equipment. Only patients scheduled for bronchial provocation tests were instructed to discontinue short‐acting (SABA) and long‐acting β2‐agonists (LABA) for 8–24 h. Informed written consent was collected from all participants at enrolment.

Continuous and dichotomized variables were compared across the Fe_NO_ profiles using the Kruskal–Wallis and the Chi^2^ tests (Stata/IC 16.1). Statistical significance level was set at a *p*‐value < 0.05.

The median (Q1, Q3) pre‐ and post‐BD Fe_NO_ levels (parts per billion) were 20.0 (13.0, 36.5) and 20.5 (12.0, 36.5) (*n* = 236), respectively (*p* = 0.079) (Online Repository 1). While only 107 (63.1%) withheld BD use prior to the test, pre‐ (19.0 [14.0, 34.0] vs. 22.0 [13.0, 40.0], *p* = 0.545) and post‐BD Fe_NO_ levels (19.0 [13.0, 34.0] vs. 21.0 [12.0, 41.0], *p* = 0.686) were similar across participants with and without prior BD use. In relation to pre‐ and post‐BD Fe_NO_ levels, 96 (40.7%) had a change ≤ −10% and ≤ +10% (Fe_NO_ =) (22.0 [15.0, 42.0] vs. 21.0 [14.5, 41.5], *p* = 0.470), 73 (30.9%) an increase > +10% (Fe_NO_+) (19.0 [11.0, 43.0] vs. 28.0 [16.0, 50.0], *p* < 0.001) and 67 (28.4%) a decrease > −10% (Fe_NO_–) (18.0 [13.0, 27.0] vs. 14.0 [9.00, 22.0], *p* < 0.001) (Online Repository 2).

When comparing the Fe_NO_ =, Fe_NO_+ and Fe_NO_– profiles, the proportion of patients with prior BD use was similar. Regarding spirometry, neither forced expiratory volume at first second (FEV_1_), nor in FEV_1_/forced vital capacity (FVC) differed between the three groups. Correspondingly, background characteristics, disease control, biomarker levels and medication use were similar. However, compared to the Fe_NO_= and Fe_NO_+ profiles, the Fe_NO_– profile to a lesser extent was treated with SABA. Similarly, LABA and inhaled corticosteroids (ICS) tended to be less common in the same Fe_NO_ profile; however, it was not statistically significant (Table 1). Similarly, in participants with prior BD use (*n* = 129), a pre‐BD FEV_1_ < 80% predicted (*n* = 126), a positive post‐BD FEV_1_ change > 5% (*n* = 115) and current non‐smokers (*n* = 200), SABA, LABA and/or long‐acting muscarinic agonists (LAMA) treatment were less common in the Fe_NO_– profile (Online Repository 3–9).

**TABLE 1:** Background characteristics, asthma medication use, disease control, biomarker levels and lung function indices by Fe_NO_ profiles (*n* = 236).

	All participants (*n* = 236)	Fe_NO_ = profile (*n* = 96)	Fe_NO_+ profile (*n* = 73)	Fe_NO_– profile (*n* = 67)	*p* *
	*n* (%) or median (q1, q3)	*n* (%) or median (q1, q3)	*n* (%) or median (q1, q3)	*n* (%) or median (q1, q3)	
Background characteristics					
Female sex (*n* = 236)	149 (63.1%)	60 (62.5%)	44 (60.3%)	45 (67.2%)	0.690
Age, years (*n* = 236)	56 (42, 65)	57.0 (42.5, 65.0)	56.0 (42.0, 66.0)	54.0 (42.0, 66.0)	0.990
Current smoker (*n* = 236)	36 (15.3%)	48 (50.0%)	32 (43.8%)	35 (52.2%)	0.579
Pack‐years (*n* = 220)	0.0 (0.0, 19.5)	0.0 (0.0, 23.0)	0.0 (0.0, 10.0)	0.0 (0.0, 20.0)	0.361
Asthma medication use					
Biologicals (*n* = 236)	75 (31.8%)	32 (33.3%)	25 (34.3%)	18 (26.9%)	0.589
OCS (*n* = 233)	22 (9.44%)	8 (8.42%)	10 (13.7%)	4 (6.15%)	0.289
ICS (*n* = 233)	158 (67.8%)	68 (71.6%)	53 (72.6%)	37 (56.9%)	0.086
LABA (*n* = 233)	159 (68.2%)	69 (72.6%)	53 (72.6%)	37 (56.9%)	0.070
LAMA (*n* = 233)	32 (13.7%)	14 (14.7%)	7 (9.59%)	11 (16.9%)	0.419
SABA (*n* = 233)	145 (62.2%)	64 (67.4%)	52 (71.2%)	29 (44.6%)	**0.003**
SAMA (*n* = 233)	66 (28.3%)	29 (30.5%)	21 (28.8%)	16 (24.6%)	0.714
LTRA (*n* = 233)	60 (25.8%)	27 (28.4%)	16 (21.9%)	17 (26.2%)	0.631
Any medication use (*n* = 233)	204 (87.6%)	85 (89.5%)	67 (91.8%)	52 (80.0%)	0.085
Asthma disease control					
ACQ‐6 (*n* = 207)	1.50 (0.67, 2.50)	1.67 (0.67, 2.67)	1.67 (0.67, 2.50)	1.50 (0.83, 2.33)	0.763
ACQ‐7 (*n* = 208)	1.71 (0.86, 2.57)	1.86 (0.86, 2.71)	1.86 (0.86, 2.57)	1.64 (1.0, 2.43)	0.769
ACT (*n* = 209)	17.0 (12.0, 21.0)	15.0 (12.0, 21.0)	18.0 (12.5, 21.0)	17.0 (13.0, 21.0)	0.359
Number of exacerbations (*n* = 233)	0.0 (0.0, 1.0)	0.0 (0.0, 1.0)	0.0 (0.0, 1.0)	0.0 (0.0, 1.0)	0.528
Inflammatory blood biomarkers					
Blood eosinophils, 10^9^/L (*n* = 211)	1.90 (1.00, 3.40)	2.15 (1.25, 3.40)	1.70 (0.90, 4.30)	1.80 (1.0, 2.80)	0.519
Total IgE > 100, kU/L (*n* = 183)	87 (47.5%)	36 (48.7%)	32 (55.2%)	19 (37.3%)	0.169
Lung function					
BD use prior to the test (*n* = 236)	129 (54.7%)	59 (61.5%)	37 (50.7%)	33 (49.3%)	0.218
Pre‐BD Fe_NO_, ppb (*n* = 236)	20.0 (13.0, 36.5)	22.0 (15.0, 42.0)	19.0 (11.0, 43.0)	18.0 (13.0, 27.0)	0.276
Post‐BD Fe_NO_, ppb (*n* = 236)	20.5 (12.0, 36.5)	21.0 (14.5, 41.5)	28.0 (16.0, 50.0)	14.0 (9.0, 22.0)	**< 0.001**
% change in Fe_NO_ (*n* = 236)	0.0 (−12.3, 13.3)	0.0 (−4.26, 4.72)	18.2 (14.3, 25.0)	−20.8 (−30.0, −15.0)	**< 0.001**
Pre‐BD FEV_1_, % pred (*n* = 233)	78.0 (63.5, 89.5)	78.0 (67.5, 88.0)	78.0 (66.0, 91.0)	78.0 (54.0, 89.0)	0.622
Post‐BD FEV_1_, % pred (*n* = 233)	83.0 (68.5, 94.0)	83.5 (70.5, 93.0)	85.0 (72.0, 95.0)	83.0 (59.0, 92.0)	0.497
% change in FEV_1_ (% pred) (*n* = 233)	4.79 (1.69, 9.19)	5.35 (2.63, 10.3)	4.72 (0.0, 8.33)	4.12 (1.45, 7.41)	0.140
Pre‐BD FEV_1_/FVC (*n* = 235)	0.73 (0.64, 0.80)	0.73 (0.64, 0.80)	0.73 (0.64, 0.81)	0.73 (0.65, 0.80)	0.930
Post‐BD FEV_1_/FVC (*n* = 235)	0.76 (0.66, 0.83)	0.76 (0.67, 0.82)	0.74 (0.68, 0.83)	0.76 (0.65, 0.84)	0.971
% change in FEV_1_/FVC (*n* = 235)	2.56 (0.0, 6.10)	2.83 (0.0, 5.92)	2.33 (0.55, 5.89)	2.30 (0.0, 6.41)	0.839

*Note:* Bold *p*‐values denote significant differences between the Fe_NO_ =, Fe_NO_+, and Fe_NO_– profiles.

Abbreviations: ACQ, asthma control questionnaire; ACT, asthma control test; BD, bronchodilator; Fe_NO_, fraction of exhaled nitrogen oxide; FEV_1_, forced expiratory volume at 1st second; FVC, forced vital capacity; ICS, inhaled corticosteroids; IgE, immunoglobulin E; kU/L, kilounits per litre; LABA, long‐acting β2‐agonists; LAMA, long‐acting muscarinic agonists; LTRA, leukotriene receptor agonist; *n*, number; OCS, oral corticosteroid; ppb, parts per billion; pred, predicted; q1, 1st quartile; q3, 3rd quartile; SABA, short‐acting β2‐agonists; SAMA, short‐acting muscarinic agonists.

* Chi^2^ test or Kruskal–Wallis test.

Our first finding was the confirmation of the post‐BD Fe_NO_ =, Fe_NO_+ and Fe_NO_– profiles in asthma, and that these patterns appeared stable irrespective of prior BD use. Second, patients with the Fe_NO_– profile were treated with asthma medications to a lesser extent.

With no significant differences in pre‐ and post‐BD Fe_NO_, our results correspond with previous studies showing a minor or no effect of BD [4, 5, 6]. Nevertheless, three distinct post‐BD Fe_NO_ profiles were identified, consistent with the papers by Michils et al. (*n* = 67) [4] and Demey et al. (*n* = 30) [7]. However, our study population was larger, overall exhibiting better disease control, preserved lung function, less obstruction and smaller response to BD, thus contributing with new knowledge. Based on ventilation distribution tests and theoretical models, Michils et al. [4] suggested that these profiles likely could be explained by site of obstruction relief: central (Fe_NO_ =), pre‐acinar (Fe_NO_+) and intra‐acinar (Fe_NO_–) airways, respectively. As no such measurements were available, we can only speculate on this aspect.

While clinical characteristics overall were similar across the Fe_NO_ profiles, SABA use was less common in the Fe_NO_– profile. In the same profile, LABA and ICS use tended to be less common, however not statistically significant. This may suggest that this group might have less symptoms and therefore have less medication, or that a lower degree of treatment might be associated with more obstruction at intra‐acinar airway level.

Relying on normal subject variability post‐BD, the potentially arbitrary threshold (±10%) used to define the Fe_NO_ profiles may be a limitation. However, in a test–retest, Michils et al. reported acceptable reproducibility [4], and an independent research group recently validated these cut‐offs in relation to small airway dysfunction in adult asthma (*n* = 92) [8]. No ventilation distribution tests were performed and is a major limitation. However, the main purpose of this study was not to validate the meaning of the different Fe_NO_ behaviours, as we already have done this in three other studies; both in COPD and asthma patients [4, 7, 9]. With a relatively small, homogeneous, monocentre sample of rather well‐controlled asthmatics, in which only 63.1% withheld BD use prior to the test, the generalizability of our findings is limited to similar populations, overall affecting the reliability of our findings.

Based on a larger and completely independent cohort of asthma patients, this study confirmed the three distinct Fe_NO_ profiles post‐BD previously described. These profiles appear unrelated to previous BD use, and may reflect the extent of airway obstruction and/or the site of obstruction relief. While no obvious differences in clinical characteristics across these patterns were seen, we are first to describe that the Fe_NO_– profile was associated with infrequent bronchodilator use, suggesting a milder asthma phenotype or undertreatment. Thus, assessing Fe_NO_ both pre‐ and post‐BD may provide insights into asthma disease burden and in theory help identify the location of airway obstruction. With this, Fe_NO_ may prove useful in clinical assessment of asthma control and/or treatment evaluation. However, research is limited and further studies are needed to establish the clinical significance and its potential in clinical use.

## Author Contributions

Martin Färdig contributed to conception and design of the study, data curation, analysis and interpretation of data, manuscript writing and editing. Alain Michils, Renaud Louis, Mare Sabbe, Alain Van Muylem and Florence Schleich contributed to conception and design of the study, data collection, interpretation of data and critically revised the manuscript. Andrei Malinovschi contributed to conception and design of the study, data curation, interpretation of data and critically revised the manuscript. All listed authors approved the final version of the manuscript before submission and agreed to be accountable for all aspects of the work.

## Ethics Statement

The study was conducted according to the guidelines of the Declaration of Helsinki, and multicentre approval was permitted on December 12, 2018, by the ethics committee of Erasme University Hospital (P2018/481), as well as by the ethics committee of the University Hospital of Liège (2018/311) on December 4, 2018. Informed written consent was collected from all participants at enrolment.

## Conflicts of Interest

The authors declare no conflicts of interest.

## Data Availability

The data that support the findings of this study are available on request from the corresponding author. The data are not publicly available due to privacy or ethical restrictions.
